# Feline Neural Progenitor Cells II: Use of Novel Plasmid Vector and Hybrid Promoter to Drive Expression of Glial Cell Line-Derived Neurotrophic Factor Transgene

**DOI:** 10.1155/2012/604982

**Published:** 2012-03-20

**Authors:** X. Joann You, Jing Yang, Ping Gu, Chee Gee Liew, Henry J. Klassen

**Affiliations:** ^1^The Gavin Herbert Eye Institute, School of Medicine, University of California, Irvine, Hewitt Hall, Rm. 2014, 843 Health Sciences Road, Irvine, CA 92697-1705, USA; ^2^Department of Ophthalmology, Shanghai Ninth People's Hospital, Shanghai Jiao Tong University, School of Medicine, Shanghai 200011, China; ^3^Stem Cell Center, Department of Cell Biology and Neuroscience, University of California Riverside, Riverside, CA 92521, USA; ^4^University of California, Irvine, Hewitt Hall, Rm. 2014, 843 Health Sciences Road, Irvine, CA 92697-1705, USA

## Abstract

Sustained transgene expression is required for the success of cell transplant-based gene therapy. Most widely used are lentiviral-based vectors which integrate into the host genome and thereby maintain sustained transgene expression. This requires integration into the nuclear genome, and potential risks include activation of oncogenes and inactivation of tumor suppressor genes. Plasmids have been used; however lack of sustained expression presents an additional challenge. Here we used the pCAG-PyF101-eGFP plasmid to deliver the human GDNF gene to cat neural progenitor cells (cNPCs). This vector consists of a CAGG composite promoter linked to the polyoma virus mutant enhancer PyF101. Expression of an episomal eGFP reporter and GDNF transgene were stably maintained by the cells, even following induction of differentiation. These genetically modified cells appear suitable for use in allogeneic models of cell-based delivery of GDNF in the cat and may find veterinary applications should such strategies prove clinically beneficial.

## 1. Introduction

Transplantation of neural stem or progenitor cells for treatment of neurodegenerative diseases is an approach that has shown considerable promise in a variety of animal models (as reviewed by [1–4]). One region of the central nervous system (CNS) where particular progress has been notable is the retina, where cells of this type have been shown to integrate into immature neonatal [5], as well as mature degenerative [6] host rats, and exhibit morphological profiles suggestive of resident local neurons. Studies of this type have also been extended to nonrodent species, including the immature Brazilian opossum [7] and the dystrophic Abyssinian cat [8]. Throughout this work, transplantation of neural progenitor cells (NPCs) to the retina has been shown to be well tolerated in allogeneic models [9] and even some xenogeneic situations [7]. Survival of NPCs as grafts does not therefore routinely require systemic immune suppression, although exceptions certainly exist, as has been clearly documented [10, 11].

The results of the above work with NPC transplantation to the eye, together with a substantial volume of related studies, have helped to nurture enthusiasm for the translational development of this technology. The goal of these efforts is the treatment of a range of conditions affecting the retina, for which current clinical outcomes frequently leave room for improvement and many of which remain incurable, despite impressive recent pharmacological advances. The abilities of NPCs to be expanded in culture, integrate into retinal tissue, survive without immune suppression, and differentiate in presumptive retinal cell types all represent favorable characteristics for a donor cell type to possess. However, the apparent inability of NPCs to generate photoreceptor cells [6], at least in sizeable numbers [7], does restrict their use as a means of cell replacement in the retina. This constraint does not mitigate their potential effectiveness in an alternate role, namely, as delivery vehicles for neuroprotective cytokines.

Neurotrophic factors contribute greatly to promoting cell survival of specific neurons in the CNS. Among the most potent for this purpose are glial cell line-derived neurotrophic factor (GDNF), brain-derived neurotrophic factor (BDNF), and ciliary neurotrophic factor (CNTF). Among these, GDNF is known to be antiapoptotic [12] in the brain [13, 14], spinal cord [15], and retina [16–19]. Receptors for GDNF are known to be expressed by cells of the mature retina [16, 19, 20]. Several types of stem and progenitor cells have been genetically modified to overexpress neurotrophic factors, resulting in enhanced levels of growth factor secretion and an enhanced ability to rescue retinal neurons and preserve visual function following transplantation to animal models of retinal injury and disease [21]. Neural progenitor cells derived from the human cerebral cortex that had been genetically modified to over-express GDNF showed considerable efficiency in delaying neural degeneration [22], and the same strategy has been investigated in the retina [23].

Viral vectors have been widely used for transgene delivery [24] and are currently regarded as the most efficient method. However their use is limited due to safety issues, DNA loading capacity, and difficulties in scale-up for production. An alternate approach that does not require integration of the gene into the genome and therefore avoids the risk of insertional mutagenesis is the use of autonomously replicating plasmids or episomes (as reviewed by [25]). In episomally replicating plasmids, sequences of incorporated DNA (generally viral) enable the plasmid to replicate extrachromosomally. This poses several advantages over integrating systems: (1) the transgene cannot be interrupted or subjected to regulatory constraints that often occur with integration into cellular DNA; (2) higher transfection efficiency can be obtained than with chromosome-integrating plasmids; (3) episomes display a low mutation rate and tend not to rearrange [26]; (4) episomally replicating systems have the ability to transfer larger amounts of DNA [27].

In the present study, we explore the efficiency of non-viral plasmid vector pCAG-PyF101-eGFP mediated gene delivery in NPCs of feline origin. This plasmid consists of the CAGG composite promoter derived from the fusion of the human cytomegalovirus major immediate early enhancer (HCMV-MIE), chicken *β*-actin promoter, and rabbit globin intron sequence [28] that drives the expression of a transgene linked to a downstream internal ribosomal entry site (IRES) and a drug selection cassette. This plasmid has previously been shown to resist gene silencing in murine and human embryonic stem cells [29, 30]. Importantly, the inclusion of a virus mutant polyoma enhancer sequence, PyF101, ensures continuous transgene expression in the absence of drug selection [29]. To assess whether an efficient transgene delivery and persistence transgene expression can be achieved in neural progenitor cells, we first overexpressed the eGFP reporter gene in cNPCs as a proof of principle. Here we show that eGFP can be efficiently delivered to cNPCs using regular transfection methods. These cells continued to express eGFP for more than 60 days without significant loss of the eGFP expression. We then overexpressed GDNF in cNPCs and showed that transgenic cNPCs produced elevated levels of GDNF in the culture media and retained their identity of neural progenitors.

## 2. Material and Methods

### 2.1. Culture of Cat Neural Progenitor Cells (NPCs)

Primary cNPCs were derived from the brains of 47-day cat fetuses as previously described [8]. For the present work, a frozen sample of cNPCs at passage 9 (P9) was thawed and cultured in Ultraculture medium (Lonza, Walkersville, MD), supplemented with 2 mM L-glutamine, 20 ng/mL epidermal growth factor (human recombinant EGF; Invitrogen, Carlsbad, CA), and 20 ng/mL basic fibroblast growth factor (human recombinant bFGF; Invitrogen). The complete Ultraculture-based medium is designated UM. Cells were passaged every 3-4 days.

### 2.2. Transfection of cNPCs with pCAG-PyF101-eGFP

The pCAG-PyF101-eGFP plasmid was purified using a QIAprep spin maxiprep kit (QIAGEN, Valencia, CA). Plasmid transfection was performed using Lipofectamine 2000 reagent (Invitrogen, Carlsbad, CA) following the manufacturers' instructions. Briefly, 2 million cNPCs were seeded into a T25 culture flask and allowed to grow overnight. Separately, 8 mg of plasmid DNA and 20 uL of Lipofectamine 2000 reagent were each individually diluted in 0.5 mL of Ultraculture-based proliferation medium (UM, as described above), mixed, and allowed to stand for 5 min. The diluted DNA and Lipofectamine 2000 were then combined, mixed, and allowed to stand for another 20 min. Meanwhile, the T25 flask containing cNPCs was washed once with fresh UM, which was replaced entirely with another 2 mL of fresh UM. The transfection mixture was added dropwise and mixed. The flask was kept in a cell culture incubator under standard conditions (37°C, 5% CO_2_) for 48 h. Cells were subsequently reseeded into two T25 flasks, and selection was performed via the addition of 1.0 ug/mL puromycin to the culture medium for a duration of at least two weeks. The expression of eGFP by transfected cNPCs was monitored by fluorescence microscopy and photographed daily.

### 2.3. pCAG-PyF101-GDNF Plasmid Construction and Transfection of cNPCs

To construct pCAG-PyF10-GDNF, the original plasmid pCAG-PyF101-eGFP was digested with NotI/XhoI. The digested eGFP fragments were excised and replaced with human GDNF BstbI/NotI fragment from pEX-Z0010-Lv31 (GeneCopoeia, Germantown, Maryland) by blunt ends ligation. pCAG-PyF101-GDNF transfection of cNPCs was performed in the same manner used for pCAG-PyF101-eGFP, as described above.

### 2.4. Cell Growth Assessment Using IncuCyte Live Cell Monitoring System

The growth properties of pCAG-PyF101-GDNF transfected and nontransfected cNPCs were assessed by culturing cells under proliferation conditions in ultraculture-based medium (UM). Cells of identical passage number (P17) were seeded in a 24-well plate at a density of 40,000 cells/well. Cells were photographed and counted at 24 h intervals, based on 2 distinct measures, namely, nuclear counts and percentage confluency. Both parameters were measured using an IncuCyte (Essen Instruments, Ann Arbor, Michigan) live cell monitoring system installed within the incubator. For nuclear counts, triplet wells were labeled using the nuclear-specific fluorescent dye Vybrant DyeCycle Green Stain (Molecular Probes, Invitrogen), which binds to double-stranded DNA in viable cells. The dye was added to cultures 30 min prior to assessment and nuclear profiles counted using the proprietary IncuCyte program at 24, 48, 72, and 96 h after seeding of cells. Cells were also measured by percentage confluency at the same time points, again using the proprietary IncuCyte program.

### 2.5. Differentiation of cNPCs In Vitro

To differentiate cNPCs, the cells were cultured in ultraculture-based medium containing 10% FBS but not recombinant growth factors (UM-FBS) for a period of 5–15 days, prior to further analysis via FACS, ICC, or ELISA.

### 2.6. Fluorescence-Activated Cell Sorting (FACS) Analysis

For FACS analysis, puromycin-selected pCAG-PyF101-eGFP transfected and nontransfected cNPCs were seeded in T25 flasks (0.25 million cells/flask) and cultured for 7 days in either UM or UM-FBS. Cells were then harvested and filtered through cell strainer caps (35-*μ*m mesh) to obtain a single-cell suspension (approximately 10^6^ cells/mL). Cells were analyzed in an automated manner using a FACSAria (BD Biosciences, San Diego, CA) and FACSDiva software (BD Biosciences), without need for cell labeling or nuclear dyes. The GFP fluorochrome was excited by this instrument's standard 488 nm laser, while fluorescence was detected using a 510/20 filter.

### 2.7. ELISA Analysis

Plasmid pCAG-PyF101-GDNF transfected cNPCs were cultured in UM or UM-FBS, and the effects of differentiation on transgene expression were assessed by ELISA. In the case of undifferentiated cNPC controls, cells were seeded in T25 culture flasks in UM and allowed to grow for one or three days. At the end of days 1 and 3, culture media were replaced with 4 mL of fresh UM. Twenty four hours later, conditioned media were collected, and cultured cells were counted and collected at days 2 and 4 for ELISA analysis. For differentiated cNPCs, cells were seeded in T75 culture flask and cultured in UM-FBS for 7 days. Fresh UM-FBS medium was exchanged at day 6, and, 24 h later, conditioned medium was collected for ELISA analysis. Cells were also counted and collected for ELISA.

ELISA analysis was performed using a human GDNF DuoSet ELISA kit and protocol from R and D systems (Minneapolis, MN). Wells of microtiter plates were coated (overnight, room temperature) with 2 *μ*g/mL of GDNF capture antibody in 100 *μ*L of coating buffer (0.05 M Na_2_CO_3_, 0.05 M NaHCO_3_, pH 9.6). Blocking was performed with 1% BSA in PBS for 1 h at room temperature. Samples (100 *μ*L) were loaded in triplicates and incubated for 2 h at room temperature, followed by the addition of 100 *μ*L antibody detection antibody (0.1 *μ*g/mL) for additional 2 h at room temperature. HRP-conjugated streptavidin (1 : 200) in blocking buffer was added (20 min, room temperature), and the reaction was visualized by the addition of 100 *μ*L of substrate solution and incubation for 20 min. The reaction was stopped with 50 *μ*L H_2_SO_4_, and absorbance at 450 nm was measured with reduction at 540 nm using an ELISA plate reader. Plates were washed five times with washing buffer (PBS, pH 7.4, containing 0.1% (v/v) Tween 20) after each step. As a reference for quantification, a standard curve was established by a serial dilution of recombinant GDNF protein (31.25 pg/mL–2.0 ng/mL).

### 2.8. Immunocytochemistry

Transfected and nontransfected cNPCs were seeded on 4-well chamber slides (Nalge Nunc International, Rochester, NY) and allowed to grow for 3–5 days. Cells were fixed with freshly prepared 4% paraformaldehyde (Invitrogen, Carlsbad, CA) in 0.1 M phosphate-buffered saline (PBS) for 20 min at room temperature and washed with PBS. Cells on slides were incubated in antibody blocking buffer (PBS containing 10% (v/v) normal goat serum (NGS) (BioSource, Camarillo, CA), 0.3% Triton X-100, 0.1% NaN3 (Sigma-Aldrich, Saint Louis, MI)) for 1 h at room temperature. Slides were then incubated with primary antibodies at proper dilutions (Table 1) overnight at 4°C. The next morning, after washing, slides were incubated in fluorescent-conjugated secondary antibody (Alexa Fluor546-goat anti-mouse/rabbit, 1 : 800 in PBS, BD) for 1 h at room temperature. After an additional wash, slides were mounted using DAPI-containing Vectashield Hard Set Mounting Medium (Vector laboratories, Burlingame, CA) for 20 min at room temperature. Negative controls for immunolabeling were performed in parallel using the same protocol but without primary antibody. Fluorescent labeling was judged as positive only with reference to the negative controls. Immunoreactive cells were visualized and imaged using a Nikon fluorescent microscope (Nikon, Eclipse E600, Melville, NY).

## 3. Results

### 3.1. Ubiquitous and Constitutive Reporter Gene Expression in eGFP-Transfected cNPCs

Expression of eGFP in pCAG-PyF101-eGFP transfected cNPCs was monitored by fluorescence microscopy to assess the transfection efficiency of the plasmid vector. Transfected cells began to exhibit eGFP-related fluorescence at 18 h following-transfection. The percentage of cells expressing eGFP reached approximately 40% by day 3. At the end of day 3, cells were reseeded, and puromycin (1 ug/mL) was added to the culture medium in order to select for stable transfectants. After 2 weeks of drug selection, a preponderance of the surviving cells expressed eGFP (as demonstrated by FACS analysis below). Continued propagation of these cells in UM for more than 60 days showed that eGFP expression was ubiquitously retained in the cells.

### 3.2. Sustained eGFP Transgene Expression under Differentiation Conditions

**Figure 1:**
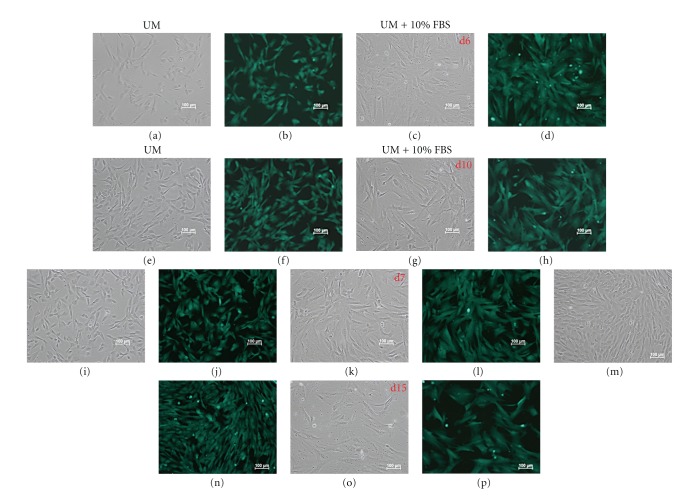
GFP expression in cNPCs transfected with pCAG-PyF101-eGFP plasmid. Transfected cNPC (passage 21) were maintained under proliferation conditions (UM) or switched to growth factor-free differentiation conditions (UM + 10% FBS) to evaluate potential loss of transgene expression. Cultures were photographed at 6, 7, 10, and 15 days. Sustained expression of green fluorescence protein (GFP) was observed for both conditions at all time points. Paired images are shown for each time point and include phase contrast (a, c, e, g, i, k, m, o) and fluorescence (b, d, f, h, j, l, n, p) micrographs of the same areas in culture.

To evaluate the influence of cellular differentiation on transgene expression, transfected cNPCs (at P21) were cultured in UM containing 10% FBS for at least two weeks. Fluorescence microscopy detected eGFP expression in almost every cell, indicating that the transfection procedure was efficient and that the pCAG-PyF101-eGFP plasmids were stably maintained and actively transcribed without significant attenuation due to cell division or growth under *in vitro* differentiation conditions (Figure 1).

**Figure 2:**
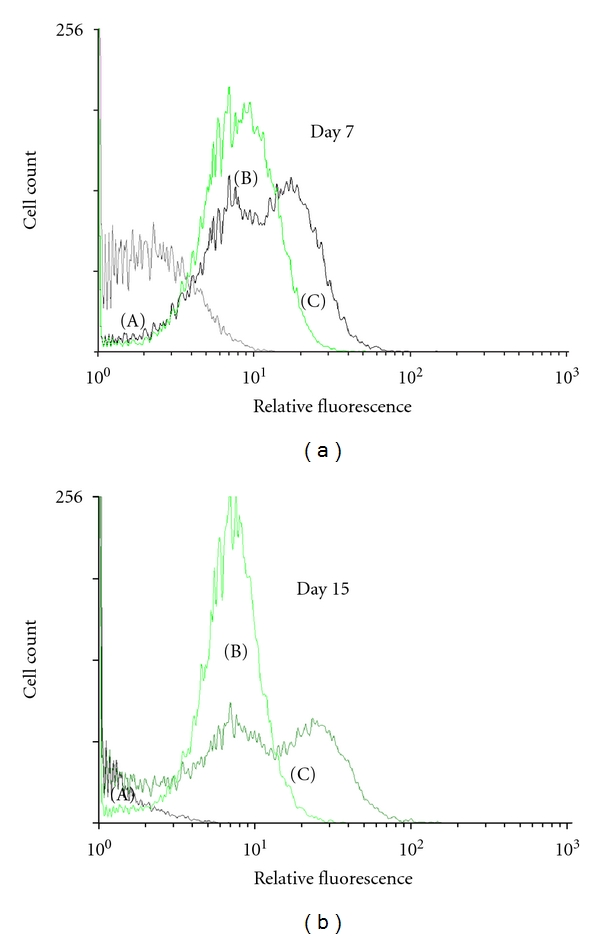
FACS analysis of GFP expression in nontransfected and transfected cNPCs. FACS analysis was performed on nontransfected and transfected cNPCs to show the expression of the GFP reporter gene. The vertical axis shows cell count, and the horizontal axis shows relative fluorescence. (a) Nontransfected cNPCp25 cultured in UM; (b) transfected cNPCp25 cultured in UM; (c) transfected cNPCp25 cultured in UM containing 10% FBS for 7 days (a) and 15 days (b).

Flow cytometric analysis was performed to further confirm eGFP expression in transfected cNPCs. Almost all transfected cNPCs cultured in either UM or UM containing 10% FBS (differentiation medium) expressed eGFP (Figure 2). Interestingly, eGFP fluorescence intensity increased in transfected cells cultured in UM containing 10% FBS. We also detected two subpopulations of transfected cells that expressed eGFP at different levels (“high” and “medium high”). This observation may relate to the heterogenous morphology and size distribution of differentiating cells, some of which are larger in size which might serve to dilute the eGFP concentration within the cell.

### 3.3. Overexpression of GDNF in cNPCs

**Figure 3:**
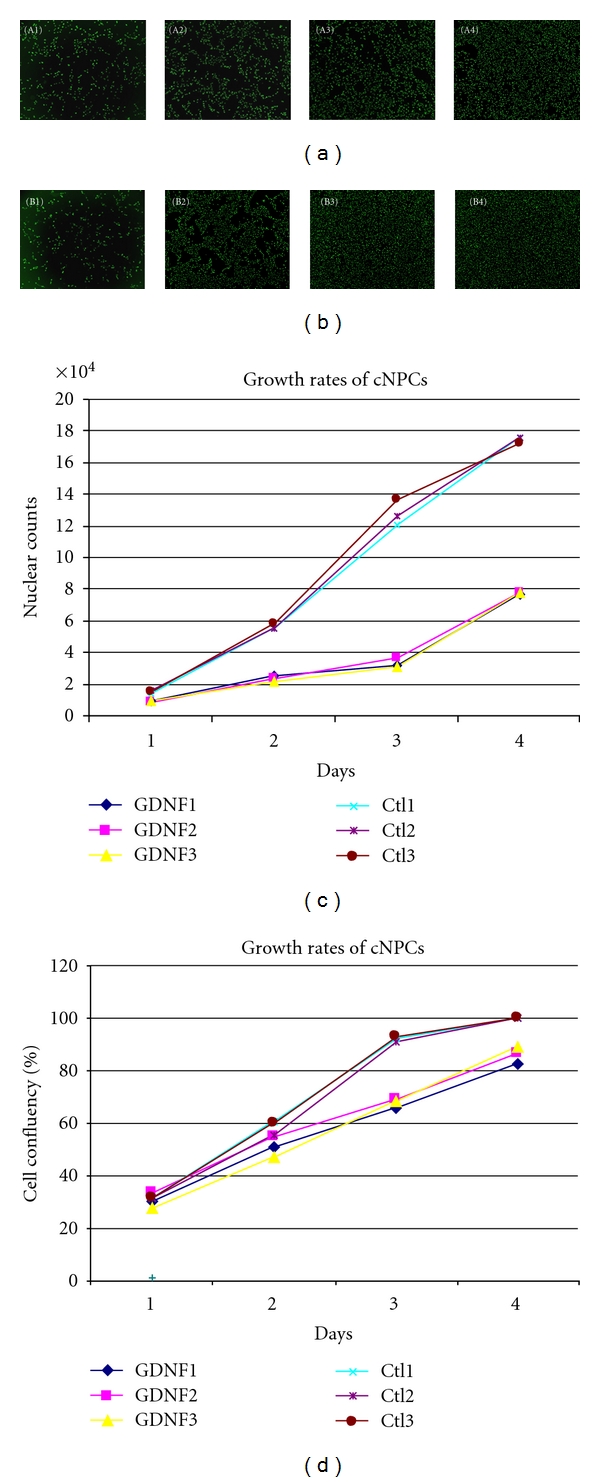
Growth of nontransfected and pCAG-PyF101-eGDNF plasmid transfected cNPCs. The fluorescence stained nuclei of (a) transfected and (b) nontransfected cNPCs. cNPCs were cultured in 24-well plate in UM for 4 days. Cells were labeled with a marker for nuclear DNA, and an IncuCyte live cell monitoring system was used to assess the growth rate of cells on each day. (a) GDNF-transfected cNPCs; A1–4: Day1–4. (b) Nontransfected cNPCs; B1–4: Day1–4. (c, d) Growth curves of GDNF-transfected and nontransfected cNPCs (labeled GDNF and Ctl, resp.) imaged and analyzed using an IncuCyte live cell monitoring system. Cells were stained with Vybrant Dycycle fluorescence nuclear-specific dye daily for 4 consecutive days (c). At the same time, cells in duplicate sets of wells without nuclear stain were measured for percentage cell confluency (d). The tight grouping of control data in (d) makes it difficult to discern Ctl1, which is present in the upper group and reaches confluence rapidly along with the other nontransfected cNPCs. IncuCyte programs were used for both analyses (c, d).

To further demonstrate the application of the pCAG vector for efficient transgene delivery beyond an eGFP reporter, we next overexpressed human GDNF in cNPCs. The effect of transduction on cellular proliferation was evaluated using an IncuCyte live cell monitoring system, allowing sequential observation of cell number in undisturbed cultures (Figures 3(a) and 3(b)). Both transfected and nontransfected cNPCs were seeded in 24-well plates in UM. Cell numbers were evaluated in terms of nuclear count and assessment of relative confluence (percentage). The resulting data showed that transfected cNPCs continued to proliferate, albeit at a slower rate than that of nontransfected cNPCs (Figures 3(c) and 3(d)). Interestingly, the monolayer cultures of the GDNF-transfected cNPCs appeared to be healthier, with fewer floating cells, less cell clumps, and minimal evidence of cell death.

### 3.4. Confirmation of Increased GDNF Protein Production by Transfected cNPCs

**Figure 4:**
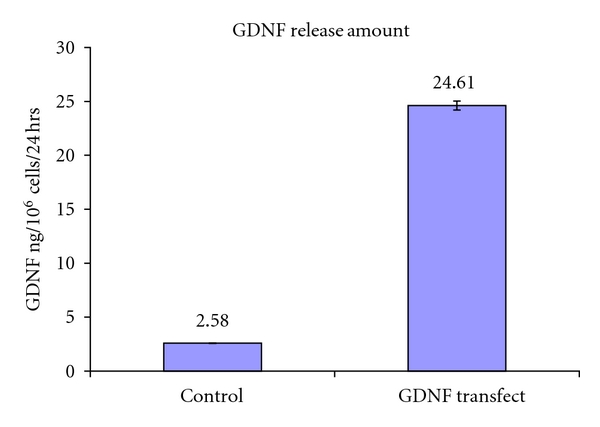
The amount of GDNF produced by nontransfected and transfected cNPCs measured by ELISA. Nontransfected and transfected cNPCs were seeded in UM. Culture media were refreshed 24 hours prior to collection of conditioned media for ELISA. Samples of culture media conditioned by transfected or control cells were analyzed to determine the level of GDNF present. Error bars = SEM.

The levels of GDNF produced by GDNF-transfected cNPCs were measured by ELISA. Cells were cultured in UM or UM containing 10% FBS, and fresh media were added 24 hours prior to collection of conditioned media for ELISA analysis. The data showed that GDNF-expressing cNPCs produced large amount of GDNF, even 60 days after initial transfection. In addition, cells cultured in UM as well as those cultured in UM containing 10% FBS produced similar amounts of GDNF (Figure 4), indicating that GDNF expression was maintained under in vitro differentiation conditions. We also determined the level of GDNF present within transfected cells. ELISA indicated that intracellular GDNF level was low (<10 ng/10^6^ cells/day, data not shown) compared to the amount of GDNF present in culture media (227–258 ng/10^6^ cells/day). Therefore the majority of GDNF produced was secreted into the culture media.

### 3.5. Immunocytochemistry Confirms GDNF and Absence of Treatment-Related Changes in cNPCs

**Figure 5:**
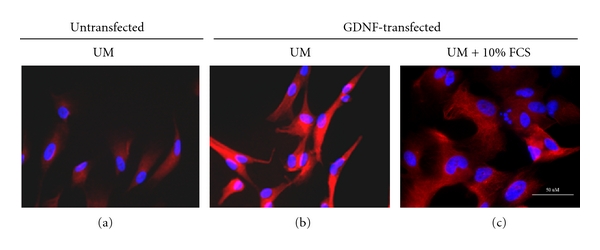
GDNF expression profiles in nontransfected and transfected cNPCp19. GDNF expression profiles were evaluated by immunocytochemistry (ICC) on cNPCs using an anti-human rabbit poly clone antibody. (a) Nontransfected cNPCs in UM; (b) pCAG- PyF101-GDNF plasmid transfected cNPCs in UM; (c) pCAG-PyF101-GDNF plasmid transfected cNPCs in UM containing 10% FBS for 5 days.

Immunocytochemical analysis was performed to confirm elevated GDNF protein expression within treated cNPCs and to evaluate the potential effects of pCAG transduction and GDNF overexpression on the ontogenetic status and lineage potential of the cells. First, an anti-GDNF antibody was used to detect GDNF protein in transfected cNPCs (Figure 5). This showed that GDNF expression was modest in nontransfected cNPCs (Figure 5(a)), but was strongly expressed in the transfected cells (Figure 5(b)). Although GDNF protein was also prominent in FBS-treated cNPCs, the fluorescence level was weaker, implying a more dilute distribution of GDNF, perhaps reflecting the larger size of the differentiating cells (Figure 5(c)).

**Figure 6:**
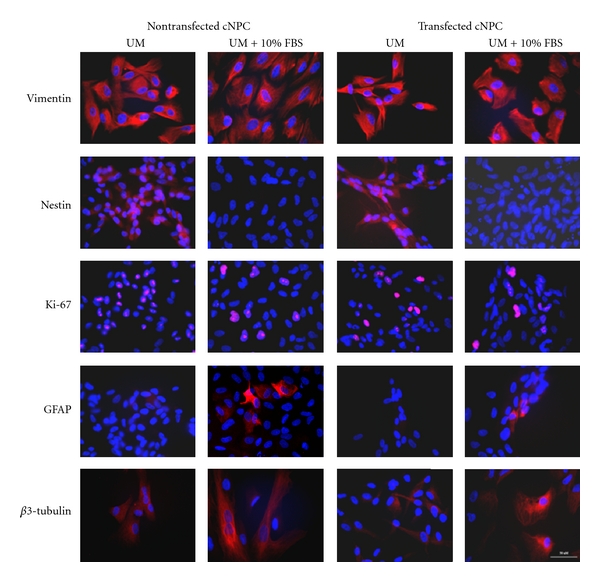
Expression profiles of marker genes in transfected cNPCs by ICC. Gene expression profiles of several neural progenitor, cell proliferation, and differentiation markers were evaluated by immunocytochemistry (ICC). Transfected cNPCs were cultured in UM, or UM containing 10% FBS, for 5 days and then immunolabeled with different epitope-specific antibodies to detect the markers shown.

Immunocytochemical analysis of a range of neural progenitor markers showed that GDNF overexpression did not significantly affect the expression of neural progenitor and proliferation markers (Figure 6). Thus, GDNF-expressing cNPCs retained their identity as neural progenitors.

## 4. Discussion

Here we report the use of a nonintegrating, plasmid-based vector to effectively transfect neural progenitor cells with an exogenous gene encoding a neuroprotective growth factor. Specifically, this plasmid vector, also containing the CAGG hybrid promoter and polyoma virus mutant enhancer PyF101a, was used to deliver the human GDNF gene to progenitor cells cultured from the fetal cat brain (cNPCs). This is one of the few studies investigating the genetic modification of NPCs derived from nonrodent, nonprimate mammalian species [24] and the first study to demonstrate the applicability of plasmid-based vector technology to feline NPCs.

Gene transfer represents a powerful tool for enhancing the desired characteristics of a therapeutic cell type. Early work exploiting transgenic reporter genes and disease models has been followed by more ambitious strategies, including cytokine delivery, immune modulation, and, more recently, cellular reprogramming [31, 32]. Nevertheless, the enthusiasm surrounding these advances has been tempered by the realization that integration of exogenous transgenes into the recipient genome can result in significant complications, including malignant transformation of cells and death of treated patients. For this reason, the use of a nonintegrating plasmid-based vector is of interest in that it might avoid the potential adverse perturbations of host cell gene regulation associated with uncontrolled alteration of the chromosomal DNA-coding sequence.

One challenge connected with the use of nonintegrating vectors is the transcriptional silencing of nonchromosomal DNA sequences by host cells. Use of the highly transcribed chicken *β*-actin [33] and its derivative composite promoter CAGG has recently gained popularity as a strategy for countering this phenomenon, providing a robust tool for deriving long-term constitutive transfectants [29, 30, 33, 34]. The findings of the current study demonstrate that, in combination, the plasmid-based vector system and CAGG promoter can effectively transfect feline NPCs. These results suggest that this method could find applicability in the delivery of various other genes of interest to feline NPCs, as well as possibly other feline cells, and immature and differentiated cells from additional mammalian species.

One interesting observation is that the GDNF-transfected cells exhibited a slower growth curve than untransfected controls. The reason for this was not delineated in the current study, but could relate to a number of considerations. One of these is that the cells were genetically manipulated and this could be deleterious in a number of ways. Another is that the cells overexpress the signaling molecule GDNF, which could in turn exert physiological influences on apoptosis or rate of proliferation. In another study with murine retinal progenitor cells [35], we showed that exogenous GDNF was antiapoptotic and did not impede proliferation, suggesting that the slower growth seen in the current study likely results from the genetic modification process or resultant protein overexpression, rather than from subsequent GDNF-induced signaling.

The cells generated and banked during this study provide a uniquely modified cell type with potential scalability. As such, these genetically modified feline NPCs could be of translational interest in the setting of veterinary applications. Further studies involving transplantation will be necessary to explore the safety and therapeutic potential of these cells. In terms of feline retinal degeneration, a suitable recipient exists in the form of the retinal dystrophic Abyssinian cat [8].

## Figures and Tables

**Table 1:** Primary antibodies used for immunocytochemistry on cNPCs.

Target	Antibody type	Reactivity in retina	Source	Dilutions
Nestin	Mouse monoclonal	Progenitors, reactive glia	BD	1 : 200
Vimentin	Mouse monoclonal	Progenitors, reactive glia	Sigma	1 : 200
Ki-67	Mouse monoclonal	Proliferating cells	BD	1 : 200
GFAP	Mouse monoclonal	Astrocytes, reactive glia	Chemicon	1 : 200
*β*3-tubulin	Mouse monoclonal	Immature neurons	Chemicon	1 : 200
GDNF	Rabbit polyclonal	Growth factor	SCBT	1 : 200
